# One-Dimensional Nanostructure Field-Effect Sensors for Gas Detection

**DOI:** 10.3390/s140813999

**Published:** 2014-07-31

**Authors:** Xiaoli Zhao, Bin Cai, Qingxin Tang, Yanhong Tong, Yichun Liu

**Affiliations:** Key Laboratory of UV Light Emitting Materials and Technology under Ministry of Education, Northeast Normal University, Changchun 130024, China; E-Mails: zhaoxiaoli19870220@163.com (X.Z.); atad2008@126.com (B.C.)

**Keywords:** gas sensors, one-dimensional nanostructures, semiconductor, field-effect transistor

## Abstract

Recently; one-dimensional (1D) nanostructure field-effect transistors (FETs) have attracted much attention because of their potential application in gas sensing. Micro/nanoscaled field-effect sensors combine the advantages of 1D nanostructures and the characteristic of field modulation. 1D nanostructures provide a large surface area-volume ratio; which is an outstanding advantage for gas sensors with high sensitivity and fast response. In addition; the nature of the single crystals is favorable for the studies of the response mechanism. On the other hand; one main merit of the field-effect sensors is to provide an extra gate electrode to realize the current modulation; so that the sensitivity can be dramatically enhanced by changing the conductivity when operating the sensors in the subthreshold regime. This article reviews the recent developments in the field of 1D nanostructure FET for gas detection. The sensor configuration; the performance as well as their sensing mechanism are evaluated.

## 1. Introduction

Due to the excellent and well-known properties of one-dimensional (1D) nanoscaled materials, interest in 1D nanostructures, such as nanowires and nanotubes, for gas detection has increased substantially in recent years [1,2]. Compared with bulk materials, 1D nanostructures have obvious advantages for gas detection as follows: (i) High surface area-volume ratio. Gas sensors involve a surface response process, so a high surface area-volume ratio favors the adsorption of more gas molecules on the sensors and accelerates the charge accumulation, improving the sensitivity and response velocity [3]; (ii) High crystalline structure. Most 1D nanostructures are single crystals, which are free of defects and grain boundaries. The nature of the single crystal is favorable for the study of the sensing mechanism; (iii) Small dimensions. The nano/microscale size of 1D nanostructures can effectively decrease the sensor size, improve the integration level, decrease the power dissipation, and lower the cost.

Most 1D nanostructure gas sensors have applied semiconductors as the sensing material. The semiconductor sensor is one of the most common used gas sensors. They have advantages such as low cost, long duration, high sensitivity, and reliability, *etc.* The semiconductor materials were mainly focused on metal oxides, such as SnO_2_, V_2_O_5_, WO_3_ and TiO_2_. Most of them operate with a two-terminal resistor mode. The field-effect transistor (FET) is another alternative device configuration for gas detection [4]. One key advantage of field-effect sensors over resistor sensors is the current modulation by the extra gate electrode [5]. The sensitivity can be dramatically enhanced by modulation of the gate electrode when operating the devices in the subthreshold regime. Besides the current, the multiple parameters, such as mobility, threshold voltage, and subthreshold slope, *etc.*, also can be used for sensing. The integration of the change of multiple parameters in the test ambient can realize the selectivity, which provides a way to overcome the challenges in gas identification for semiconductor sensors. The third advantage of the field-effect sensors is that the sensor response can be enhanced by combining them in oscillator and adaptive amplifier circuits [5]. Finally, field-effect sensors can operate at room temperature, which has advantages of low power consumption, long device lifetime, and reduced explosion hazards. These merits in field-effect sensors combined with the 1D single crystal nanostructures are therefore pushing the development of the 1D nanostructure field-effect sensors. Up to the present, a series of research works on the gas sensing behavior of 1D nanostructure field-effect sensors have been reported. Table 1 lists the performance of the reported 1D nanostructure field-effect sensors. Many kinds of materials were investigated as sensing materials for the detection of different gases, including In_2_O_3_, SnO_2_, ZnO, CuO, carbon nanotubes (CNTs), Si, InAs, indolo[3,2-b]carbazole,2,8-dichloro-5,11-dihexylindolo[3,2-b] carbazole (CHICZ) and CuPc. Among them, In_2_O_3_, SnO_2_ and CNT are the most used materials. Resistor sensors generally are operated at high temperatures of 200–600 °C in order to achieve enhanced chemical reactivity between the sensing materials and the detected gases. In comparison, most of field-effect sensors can operate at room temperature, as shown in Table 1.

The purpose of this article is to review recent developments in the field of 1D nanostructure FETs in gas sensing. The methods used to obtain the main performance parameters are presented. Four different research areas are reviewed: (i) Fundamentals of the gas sensors, which presents the basic principle of the 1D nanostructure field-effect sensors; (ii) Surface modification, which gives the most commonly used method for improved sensing performance by the functionalization of semiconductor nanowire or electrodes; (iii) Doping of nanowires, which optimizes the gas sensing by increasing the defects in nanostructures; (iv) New-type device configurations, which present new designs to improve the performance of the sensors. It is hoped that the examples presented here will encourage researchers to combine different materials or methods, or produce new designs that show improved or unique sensing properties.

## 2. Gas Sensor Performance Characteristics

Sensitivity, response time, recovery time and limit of detection (LOD) are typically reported as the main performance parameters of a 1D nanostructure field-effect sensor. The definition of the sensitivity is determined by comparing the test signal of the device before and after exposure (such as conductance, resistance, current, threshold voltage and mobility *etc.*). If the value of the test signal is decreased upon exposure to the target gas atmosphere, the sensitivity *S* is defined as *P_a_*/*P_g_* or (*P_g_* − *P_a_*)/*P_g_*, where *P_g_* is the test signal of the device in the target gas, and *P_a_* is the original signal of the sensor. If the value of the test signal upon exposure to the target gas is increased, the sensitivity is defined as *P_g_*/*P_a_* or (*P_a_* − *P_g_*)/*P_a_*. The response time (recovery time) is generally defined as the time period needed for the device to undergo a change from 10% to 90% (or from 90% to 10%) of the test signal value in equilibrium upon exposure to a target gas. In the reported literature, LOD is generally estimated by the lowest concentration of the device that can detect.

Selectivity is one of the most important parameters in gas sensors. According to the International Union of Pure and Applied Chemistry (IUPAC), the selectivity refers to the extent to which it can determine particular analytes under given conditions in mixtures or matrices, simple or complex, without interferences from other components [4]. As known to all, selectivity is one of the biggest challenges for semiconductor sensors, and so far the main efforts have been addressed to the detection of such analytes in solution [24]. One main detection mechanism of semiconductor sensor is based on the principle that the conductivity of the sensing material changes as a consequence of the interaction between the detected gas molecules and the surface complexes such as O^−^, O^2−^, H^+^ and OH^−^. The formed electron-depletion layer on the semiconductor surface decreases or increases the free carrier concentration of the material. This gives rise to the changed conductivity or threshold voltage. This process makes semiconductor sensors generally present a sensing response to all oxidizing and reducing gases. Some sensors show high sensitivity to only one analyte, which is referred to as specificity, with the 100% selectivity. To date, although some sensors are very sensitive to certain analytes, for example, NO_x_, with LOD ranging from 10 to 200 ppb, they generally suffer from low selectivity or require elevated working temperatures [25].

## 3. Progress of Micro/Nanoscaled Single Crystals Field-Effect Sensors

Field-effect sensors have been known for more than four decades. In 1975, Lundstrom *et al.* reported the first field-effect sensors [26,27]. A 10-nm-thick palladium layer was used as gate electrode and Si was used as semiconductor for the detection of hydrogen. The threshold voltage of this transistor was changed with the partial pressure of hydrogen at ambient atmosphere. They proposed a model where hydrogen molecules were adsorbed on the palladium gate, and diffused into the Pd-SiO_2_ interface forming a dipole layer. These hydrogen atoms changed the work function of the inner palladium surface and therefore affected the transistor threshold voltage.

In 2000, Dai *et al.* first fabricated a field-effect sensor based on individual single-walled carbon nanotubes (SWNTs) [20]. The Ni/Au was used as source-drain electrodes and SiO_2_ was used as the dielectric. The threshold voltage shifted towards to the negative direction of the gate voltage in NH_3_ and shifted towards to the opposite direction in NO_2_. Exposure to NH_3_ for 10 min the conductance decreased two orders of magnitude, while the conductance increased three orders of magnitude when the device was exposed to NO_2_. They ascribed the dramatic conductance changes to the change of the relative location of the Fermi level of the nanotubes. Exposure of the device to NH_3_ shifted the valence band away from the Fermi level, resulting in hole depletion and reduced conductance. Exposure to NO_2_ resulted in the Fermi level shifting closer to the valence band, enriched hole carriers in the nanotube, and enhanced conductance. Since then, 1D nanostructure field-effect sensors have attracted researchers' attention, the related devices have been designed, and their work principles and sensing properties have been studied.

### 3.1. Fundamentals of Gas Sensors

**Figure 1.**
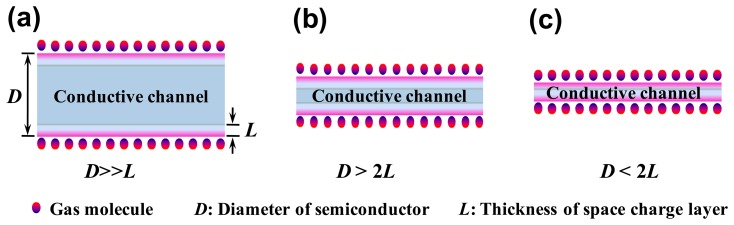
The relation between the thickness of semiconductor and the space charge layer for resistor sensor. (**a**) *D* ≫ *L*; (**b**) *D* > 2*L*; (**c**) *D* < 2*L*, *D*: the diameter of the semiconductor; *L*: the thickness of the space charge layer.

The fundamental mechanism of the semiconductor sensors is still controversial. For the semiconductor resistor sensors, the changes in conductivity are believed to be related to the formation of a space charge layer at the semiconductor surface after gas adsorption. Therefore, the ratio of the thickness of the space charge layer to the conductive channel dimension determines the sensitivity of the sensor. The higher the surface area-volume ratio, the higher the dimension ratio of the space charge region to the conductive channel, as shown in Figure 1. This explains why the adsorption of the gas molecules on nanostructures can change the conductance more dramatically. The experimental results have confirmed that the sensitivity of the sensors based on nanowires or nanoparticles is far higher than that of bulk materials [28,29].

**Figure 2.**
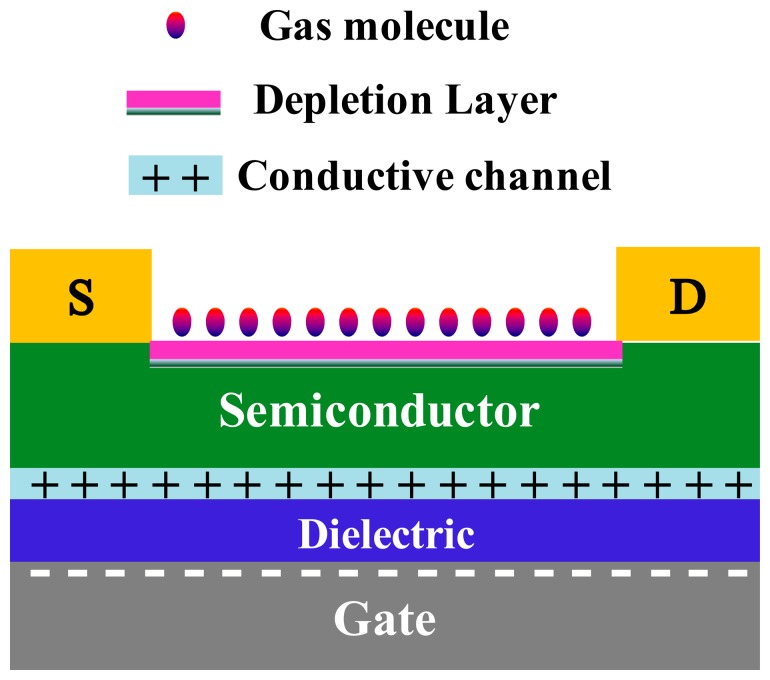
Schematic image of the interaction of the adsorbed gas molecules with FET.

For the response mechanism of the field-effect sensors, Torsi *et al.* proposed that the modulation effect of the gate electrode on the current magnified the sensor response signal [30]. Leeuw *et al.* have demonstrated the shift of the threshold voltage upon exposure to NO_2_ with n-type, p-type, and ambipolar semiconductors [31]. They ascribed the response to gate bias-induced electron trapping [28,31]. Further, by delaminating the semiconductor with adhesive tape and measuring the surface potential of the gate dielectric by scanning Kelvin probe microscopy, they showed that the trapped electrons were located at the gate dielectric [32]. Although the work principle of the field-effect sensors is controversial, it is believed that the effect of the space charge layer formed by gas adsorption on the conductive channel plays a key role. Frisble *et al.* have carried out atomic force microscope (AFM) measurements, and have confirmed that the gate voltage concentrated the conductive channel in one molecular layer at the interface between the semiconductor and dielectric [33]. On the other hand, Yamazoe *et al.* confirmed that the thickness of the space charge layer caused by gas adsorption was about a few nanometers [29]. Therefore, the size of the conductive channel is comparative to the thickness of the space charge layer in FET, as shown in Figure 2. In this case, the sensitivity of the device can be improved dramatically. For example, the reported the lowest LOD of the individual In_2_O_3_ resistor sensor is 500 ppb at the operating temperature of 400 °C [34], while the lowest LOD of the In_2_O_3_ field-effect sensor is 20 ppb at room temperature [6]. Tang *et al.* [10] recently reported that their SnO_2_ nanobelt field-effect sensors with the traditional metal electrodes indicated the sensitivity of 5.48 × 10^7^% at 50 ppb NO_2_, which was four orders higher than the reported highest resistor SnO_2_ nanobelt sensor (1900% at 100 ppb) [35], showing the dramatic effect of the gate electrode in improving the sensitivity [10]. Lu *et al.* showed that the sensitivity to oxygen of the ZnO nanowire field-effect sensor was higher with smaller-radius nanowires [13]. Moreover, the oxygen detection sensitivity could be modulated by the gate voltage. They ascribed the gate-dependence detection sensitivity to the gate modulated electron concentration in the nanowire channels. When the gate voltage is far above the threshold so that the modulation effect of the gate on the current is weak, the electron concentration in the channel is very high and the gas molecules adsorbed on the 1D nanostructure captured only a small portion of the available carriers, therefore, the relative conductance change is very small. However, when the device operates in the subthreshold regime and the FET is gated just above the threshold, the channel carriers are substantially depleted and the conductance change caused by gas adsorption becomes much more significant. This results in a high sensitivity for 1D nanostructure field-effect sensors.

As we know, most 1D nanostructure field-effect sensors work in room temperature, which makes them hardly reversible and leads to long recovery times, since the activation energy for desorption is generally higher than the thermal energy. A few research groups have demonstrated novel methods to shorten the recovery time. For example, Yang *et al.* illuminated a SnO_2_ nanobelt device with ultraviolet (UV) light of energy near the SnO_2_ band gap [36]. They found that the photogenerated carriers accelerated the desorption velocity of device on NO_2_. Lu *et al.* applied a strong negative field to refresh the ZnO nanowire field-effect sensors by an electrodesorption mechanism [14]. They proposed that a negative gate potential possibly depleted the electrons in the nanowire and reduced the number of electrons available at vacancy sites, thus lowering the chemisorption rate. The negative field drove the holes to migrate onto the surface, resulting in the discharge of gas molecules. In addition, the negative gate induced repulsive field stretches and weakened the bonding between these adatoms and adsorption sites. Yoo *et al.* applied a negative gate voltage pulse in NO_2_ and a positive gate voltage pulse in NH_3_ to refresh CNT field-effect sensors [17]. The NO_2_ molecules were physically adsorbed onto the CNT via dipole interactions, and electrons were transferred from the CNT to NO_2_ molecules. Therefore, they proposed that the negative gate voltage induced the repulsive force, which may weaken the binding between the CNT and NO_2_ molecules and accelerate their desorption.

### 3.2. Surface Modification

#### 3.2.1. Modification of Metallic Nanoparticles on Semiconductor Nanowires

Modification of metallic nanoparticles on semiconductor nanowire/nanobelt/nanotubes is the most commonly used way to improve the sensor performance for both the resistor sensors and field-effect sensors [37–45]. Noble metals, such as Pt, Ag, Pd, Au, *etc.*, are generally used as highly-effective oxidation catalysts to enhance the catalytic activity of gas sensors. Two mechanisms are considered when the 1D nanostructures are modified with metallic nanoparticles [46]. One is that the nanoparticles on the nanowire surface create the Schottky barrier-type junctions within the nanowire, resulting in the formation of electron depletion regions. The other mechanism is a “back-spillover effect”, which is a purely chemical catalytic effect when the metallic nanoparticles are dispersed on the surface of 1D nanostructures. Moskovits *et al.* compared the sensing performance of individual SnO_2_ nanowire and nanobelt gas sensors before and after functionalization of the Pd nanoparticles [46]. Pd catalyst particles were deposited *in situ* in the same reaction chamber where the sensing measurements were carried out. The *in situ* deposition and sensing characterization ensured that the observed change of sensing performance was due to the Pd functionalization rather than any variation in properties from one nanowire to another. The effect of metal deposition on the nanowire conductance was monitored throughout the deposition process by continuously measuring *I_SD_*. High resolution transmission electron microscopy (HRTEM) images confirmed that no bulk oxidation occurred for these Pd nanoparticles. Upon exposure to O_2_ and H_2_, the device showed the increased *I_SD_*. The Pd functionalized nanowire devices showed dramatically improved sensitivity and response speed, which was ascribed to the enhanced catalytic dissociation of the molecular adsorbate on the Pd nanoparticle surfaces and the subsequent diffusion of the resultant atomic species to the oxide surface.

**Figure 3.**
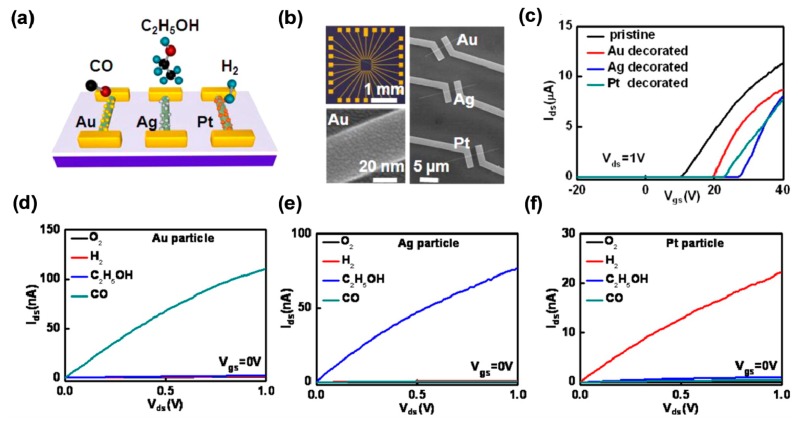
(**a**) Schematic of the sensor arrays. The surface of nanowires was decorated with Au, Ag, and Pt nanoparticles, respectively; (**b**) Optical (top left) and SEM (right) image of the chemical sensor arrays. High magnification SEM (bottom left) image of Au decorated Mg-doped In_2_O_3_ nanowire; (**c**) The transfer curves for the sensors with and without the metal decoration; (**d**–**f**) The output curves for Au, Ag and Pt decorated enhanced-mode FETs toward the gas specific detection of O_2_, H_2_, C_2_H_5_OH, and CO [8].

Another obvious advantage of functionalizing semiconductor nanowires with catalytically active metals is to achieve selectivity for gas sensors. Currently, the development of highly selective devices remains a challenge for semiconductor sensors. It has been found that the devices functionalized with different metal nanoparticles show different response characteristics to different gases [45,47–49]. Ho *et al.* used Mg-doped In_2_O_3_ nanowire enhanced-mode field-effect sensor arrays decorated with various discrete metal nanoparticles (*i.e.*, Au, Ag and Pt), to selectively distinguish among three reducing gases (e.g., CO, C_2_H_5_OH and H_2_), with high sensitivity [8]. The deep enhanced-mode FET could not detect the reducing or oxidizing gases, which provided an ideal platform to introduce the gas selectivity and sensitivity into the nanowire surface by decorating various metal nanoparticles. The schematic, optical, and scanning electron microscope (SEM) images of the Au, Ag and Pt decorated nanowire field-effect sensors are shown in Figure 3a,b. Figure 3c presents the *I_SD_*-*V_GS_* curves for the sensors with and without the metal modification. Four target gases, O_2_, H_2_, C_2_H_5_OH and CO, were added to the continuously flowing air stream at a low concentration of 100 ppm, respectively. Each type of metal nanoparticle could respond to a specific gas molecule through different catalytic reactions. Figures 3d–f present the *I_SD_*-*V_SD_* characteristics for Au, Ag and Pt-decorated FETs. The gas specific enhancement in the FET conductance was clearly demonstrated with Au, Ag and Pt nanoparticle- decorated nanowire channels for the selective detection of CO, C_2_H_5_OH and H_2_, respectively.

#### 3.2.2. Modification of Semiconductor Nanoparticles on Semiconductor Nanowires

**Figure 4.**
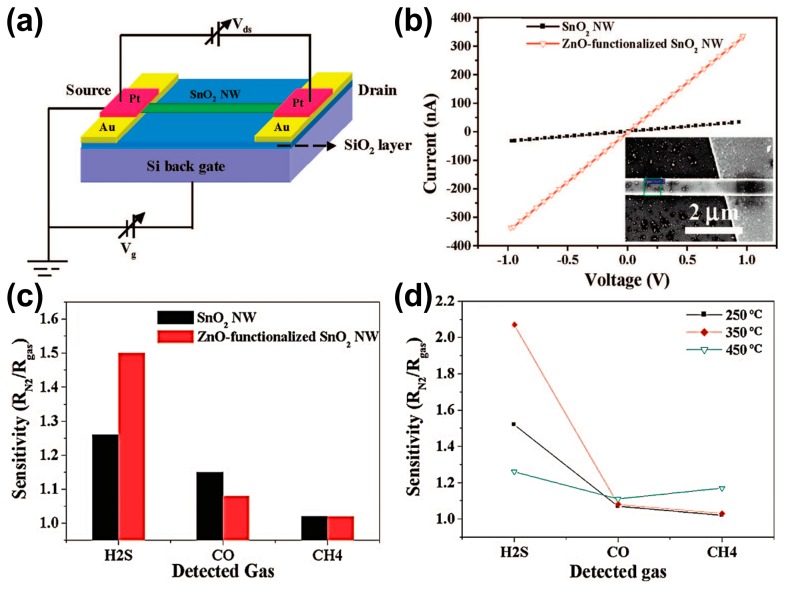
(**a**) Schematic illustration of a single SnO_2_ nanowire-based FET; (**b**) *I*-*V* curves of the single SnO_2_ nanowire device before and after ZnO deposition. The inset is the corresponding SEM image of the single SnO_2_ nanowire device after ZnO deposition; (**c**) Comparison of gas-sensing sensitivity of the pure and ZnO-functionalized SnO_2_ nanowire sensor to three detected gases; (**d**) Sensitivity to three detected gases of the ZnO-functionalized SnO_2_ nanowire sensor at different operation temperatures. The concentration of detected gases was 500 ppm, the operation temperature was 250 °C, and the fixed bias was 1 V [11].

As discussed above, surface modification is an effective method in improving sensing performance. Wang *et al.* improved the sensing properties of the SnO_2_ nanowire sensors by modification of ZnO nanoparticles. The 10-nm-thickness ZnO nanoparticles were deposited on the surface of SnO_2_ nanowires via magnetron sputtering [11]. Figure 4a is the schematic image of the device. Figure 4b shows the *I*-*V* curves of the single SnO_2_ device before and after ZnO deposition. The conductivity of SnO_2_ nanowire increased dramatically when the nanowire surface was modified with ZnO nanoparticles. Figure 4c shows the sensitivities of a SnO_2_ nanowire sensor to three detected gases (500 ppm H_2_S, CO, and CH_4_) before and after ZnO modification. It was found that the sensitivity changes were different for different gases after ZnO surface modification. After the SnO_2_ nanowire was modified with ZnO nanoparticles, the sensitivity of the device increased for H_2_S, while the sensitivity decreased for CO, and was almost unchanged for CH_4_. These results showed that the selectivity of the single SnO_2_ nanowire was improved to a certain extent by surface functionalization. Figure 4d shows the different sensitivity of the ZnO nanoparticle-modified SnO_2_ nanowire sensor at different operation temperatures upon exposure to different gases. By operating the devices at the optimized temperature, the sensitivity and selectivity of the device could be effectively improved. The device showed the highest sensitivity to H_2_S at 350 °C and to CH_4_ at 450 °C. They proposed that the formation of n-n heterojunction with an energy difference of about 0.75 eV between ZnO and SnO_2_ possibly was responsible for the enhanced selectivity [50]. At the same time the coordinate effect of two sensing materials also played a key role.

#### 3.2.3. Modification of Organic Molecules on Semiconductor Nanowires

**Figure 5.**
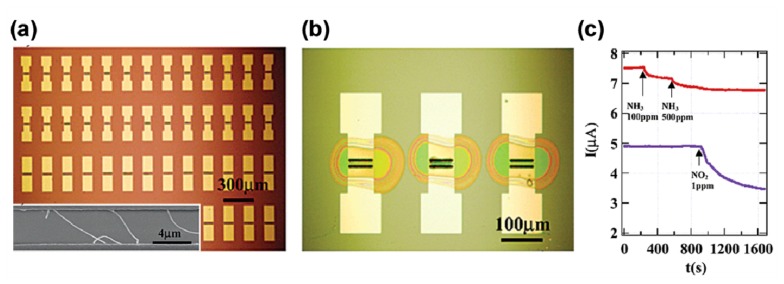
(**a**) Optical image of an array of multiple-SWNT devices. The inset is SEM image of several nanotubes bridging two opposing Mo electrodes in a device; (**b**) Optical image showing three multiple-tube (MT) devices after microspotting with droplets of polymer solutions; (**c**) Red (top) curve: a device coated with Nafion exhibited a response to 100 and 500 ppm of NH_3_ in air, and no response when 1 ppm of NO_2_ was introduced to the environment. Blue (bottom) curve: a PEI-coated device exhibiting no response to 100 and 500 ppm of NH_3_ and large conductance decrease for 1 ppm of NO_2_ [16].

Another effective method for the realization of selectivity is to modify the semiconductor with organic molecules. One way is to functionalize each semiconductor element in an array of sensors with different organic molecules, for example, polymers or silanes. Dai *et al.* functionalized multiple-nanotube array devices with two different polymers, polyethyleneimine (PEI) and Nafion, by microspotting the different solutions to position the pin over each individual device [16]. This method allowed the assembly of multiple devices could selectively detect NO_2_ and NH_3_ in gas mixtures. The morphology of the multiple-SWNT device array, a single device, and the devices after microspotting, are shown in Figure 5a,b. The PEI contains high-density amines, and renders as-grown SWNTs electron rich, and hence is high selective toward strongly electron-withdrawing molecules. Nafion is a polymer with a Teflon backbone and sulfonic acid side groups. It is sensitive to NH_3_ that tends to react with H_2_O in the environment to form NH_4_OH. As shown in Figure 5c, the Nafion-functionalized device presented a decrease of the conductance in 100 and 500 ppm NH_3_ due to NH_3_ electron donation to the p-type device reducing the majority hole carriers, while a PEI-functionalized device on the same chip did not show any response to NH_3_. When introducing 1 ppm of NO_2_ into the environment in 500 ppm NH_3_, the Nafion-functionalized device did not respond, while the PEI-functionalized device showed a decrease of the conductance. These results clearly demonstrated that multiplexed nanotube sensor arrays are promising for specific molecular detection in complex chemical environments.

Heath *et al.* selectively detected hexane and acetone vapor by modification of Si nanowires on a sensor array [21]. The Si nanowire was naturally coated by a layer of SiO_2_, which is easily modified by a lot of commercially available reagent molecules that contain silanes. They left one device unmodified, and the other three devices were chemically modified by alkane-, aldehyde- and amino-silanes using a vapor deposition method. The sensor array was fabricated as a field-effect configuration, while the gate voltage was not applied when the different gases were detected. All devices presented an increase of the conductance upon exposure to two different gases, while the magnitude of the response was different for each device that was determined by the surface chemistry.

The detection of nonpolar analytes with Si nanowire FETs remains challenging under real-world conditions. The Haick group have used ailor-made organic functionalized Si nanowire FETs and recorded the electrical response of sensors with high signal-to-noise ratios upon exposure to nonpolar analytes [51,52]. They attributed the detection of the nonpolar analytes to two indirect effects: (i) a change in the dielectric medium close to the Si nanowire surface; (ii) a change in the charged surface states at the functionality of the Si nanowire surface. The results provided clear evidence for the leading role of molecular gating in detecting nonpolar analytes and a launching pad for real-world sensing applications with Si nanowire FETs.

The “hysteresis” phenomenon of the Si nanowire FETs is generated by interactive effects [53], such as the periphery surfaces, interfaces and/or adsorbed atmosphere molecules near the charge carrier channel, which limits their performance under real-world conditions. To suppress this phenomenon, Haick *et al.* have used a series of systematically changed trichlorosilane-based organic monolayers to study the interactive effect of hysteresis and surface chemistry on a Si nanowire FET gas sensor [54]. The findings could extend the use of gated silicon nanowire gas sensors for discrimination between polar and nonpolar analytes in complex, real-world gas mixtures.

**Figure 6.**
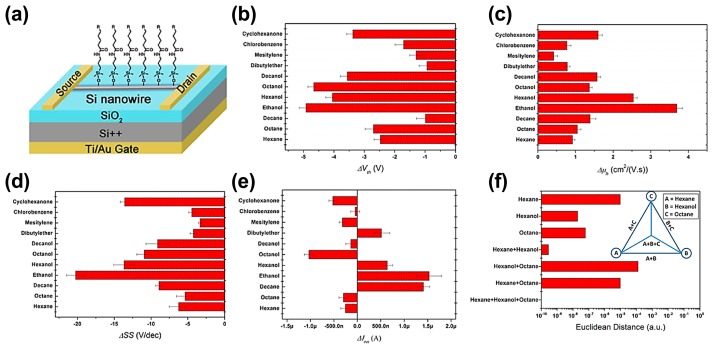
(**a**) Scheme of a molecularly modified Si nanowire field-effect sensor; (**b**–**e**) Changes in *V_th_*, μ*_h_, SS*, and *I_on_* of an electron-donating sensor on exposure to various VOCs at concentration of *p_a_*/*p_o_* = 0.08; (**f**) Euclidean distance of ANN outputs using an electron-withdrawing sensor to identify hexane, hexanol, octane and their binary and ternary mixtures. Inset: Schematics of the relationship among single VOCs and their binary and ternary mixtures in ANN outputs [56].

Although the selectivity can be improved by modifying the sensor array with different molecules or metal nanoparticles, the increased sensor number increases the power consumption and integrated size, complicating the device computation. Haick and his colleagues have used molecularly modified Si nanowire FETs to distinguish different polar and nonpolar volatile organic compounds (VOCs) in an atmosphere with background humidity (relative humidity: 40%) [55]. The combination of nanowire FET arrays has the best discriminative power between the various VOCs. Very recently, Haick *et al.* also used the multiple independent parameters of a specific molecularly modified Si nanowire FET as input for selective detection of specific VOCs [56]. Figure 6b–e presents the responses of a molecularly modified Si nanowire field-effect sensor on exposure to eleven VOCs at VOC concentration of *p_a_*/*p_o_* = 0.08 (where *p_a_* and *p_o_* are the VOC's partial pressure and vapor pressure, respectively, at a given temperature). The sensor exhibited negative responses in the threshold voltage *V_th_* and the *SS*, while it showed positive responses in μ*_h_* to all tested VOCs. In the *I_on_* response plot, the sensor functionalized with the molecule containing the COOH functional group showed positive responses to decane, ethanol, hexanol, and dibutyl ether, and negative responses to the rest of the VOCs. These multiple sensing responses by one molecularly modified Si nanowire FET created a fingerprint for each VOC, which could be used for VOC discrimination. They selected four sensing parameters, including *V_th_*, μ*_h_*, *I_on_*, and *SS*, and employed artificial neural network (ANN) models to seek selectivity towards specific VOCs. The selectivity depended on the difference in the combination of the four sensing parameters analyzed. Figure 6f shows the Euclidean distance (ED) of ANN outputs. The ED is the distance between the target vector and prediction vector, which can be used to evaluate the recognition power of the sensors. The ED values of all tested samples were below 10^−3^. By a combination of multiple parameters from one Si nanowire field-effect sensor and ANN model, each of the VOCs in the tested mixture was perfectly identified. More important, the trained ANN model successfully predicted if a certain VOC existed in a binary and even ternary VOC mixture. They also explored the effect of the functional groups and the chain length on the sensing properties of VOCs [57,58]. The change of the threshold voltage increased with the chain length of the molecular modification, while the change of relative hole mobility did not exhibit any obvious dependence on the chain length. They proposed that the electron-donating/withdrawing properties of the functional groups controlled the dipole moment orientation of the adsorbed VOCs. At the same time, the type of functional groups determined the diffusion of VOCs into the molecular layer.

#### 3.2.4. Assembly of Molecular Layer on Electrodes

**Figure 7.**
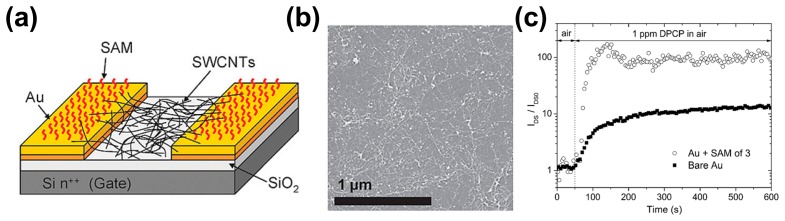
(**a**) Schematic structure of the CNT FET with functionalized gold electrodes; (**b**) SEM image of the SWNT network; (**c**) Normalized response to the CNT field-effect sensors upon exposure to DPCP vapors with bare and functionalized Au electrodes [19].

Carella *et al.* applied a self-assembled monolayer (SAM) of organophosphorus (OP) compound- sensitive molecules to functionalize the electrode of a CNT network field-effect sensor [19]. The device was fabricated with bottom gate/bottom contact geometry. Ti/Au electrodes were prepared on a Si/SiO_2_ substrate, and the functionalization was carried out by immersing the gold surface overnight in a freshly prepared 0.7 mM solution of acetone at room temperature. The schematic image of the device and the SEM image of the SWNT network are shown in Figure 7a,b. The response of CNT FET devices as a function of time at optimized V_GS_ before and after the functionalized of gold electrodes is shown in Figure 7c. The experimental results showed that the response of CNT FET to diphenylchlorophosphate/isopropyl alcohol (DPCP/IPA) vapors was highly improved after gold electrode functionalization. Pristine CNT field-effect sensors exhibited an increase of the current by a factor of ∼13, while the functionalized sensor showed a dramatically increased current by a factor ∼100. They ascribed the obviously improved response to the increased change of the Shottky barrier height at the interface between the electrode and the semiconductor after DPCP exposure, resulting in the changed the work function of the gold electrode and hence higher sensitivity for the gold electrode functionalized device. The increased Schottky barrier for holes after the gold electrode functionalization was confirmed by the decreased “ON” current, and Kelvin probe force microscopy measurements.

### 3.3. Doping of Nanowires

**Figure 8.**
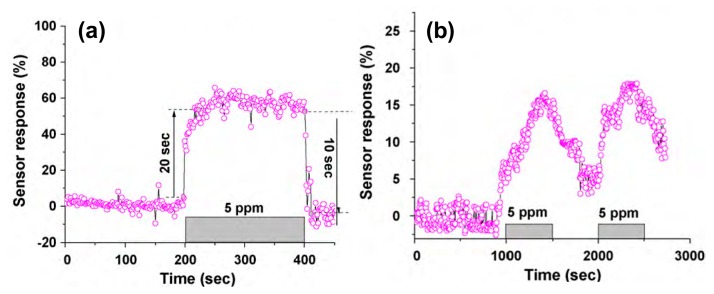
(**a**) Sensor response plot of the Zn-In_2_O_3_ nanowire sensor for single cycle of 5 ppm CO gas; (**b**) Response plot for the undoped In_2_O_3_ nanowire-FET, when exposed to 5 ppm CO gas in two cycles [7].

Doping metal oxide semiconducting nanowires is an effective strategy to improve the sensing performance. Lee *et al.* demonstrated a Zn doped In_2_O_3_ nanowire field-effect sensor for room-temperature detection of CO gas (Figure 8) [7]. The sensor was fabricated using 60 nm SiN_x_ as dielectric layer, Si as bottom gate electrode, and Cr/Au (10/50 nm) as source and drain electrodes. A good selectivity for CO gas over NO and NO_2_ could be achieved. The sensitivity of the Zn doped In_2_O_3_ nanowire sensor was found to be almost three times higher than that of the undoped nanowire sensor. The response and recovery times for the Zn doped In_2_O_3_ nanowires sensor were 20 and 10 s, respectively, which was obviously higher than those of the undoped nanowire sensor. They ascribed the enhanced sensing performance to the increase of the defect number and the modification of the Fermi level by doping. The electrical detection of the chemical species was dependent on the surface reactions between the nanowires and the chemical molecules. Most of the defects behaved as preferential adsorption sites for a gas molecule [59]. The doping induced defects improved the adsorption of gas molecules on the nanowire surface. On the other hand, doping changed the position of Fermi level of semiconductor in the energy band diagram which governed the electronic transportation between gas molecule and nanowire material. The chemical potential gradient between the adsorbed CO molecule and the nanowire was increased. This accelerated more electron transfer to reach equilibrium and therefore the faster sensor response output with higher sensitivity were obtained.

In general, SnO_2_ behaves as n-type semiconductor. Wu *et al.* fabricated p-type SnO_2_ nanowires by doping Sb [12]. Gas sensing experiments were conducted at room temperature by recording the *I*-*V* characteristics while applying a constant bias of 5 V. The doped nanowire device showed a rapid increased conductance as the device was exposed to the ethanol gas. The LOD reached 40 ppm. The response time was only ∼8.3 s for the different ethanol concentrations. The recovery time was increased from 44 to 87 s with the increased ethanol concentration in the range of 40–300 ppm. The far faster response time than the recovery time ascribed to the much lower diffusion rate of gas molecules than the charge separation rate of electron-hole pairs.

### 3.4. New-Type Device Configuration

#### 3.4.1. Gas as Dielectric

**Figure 9.**
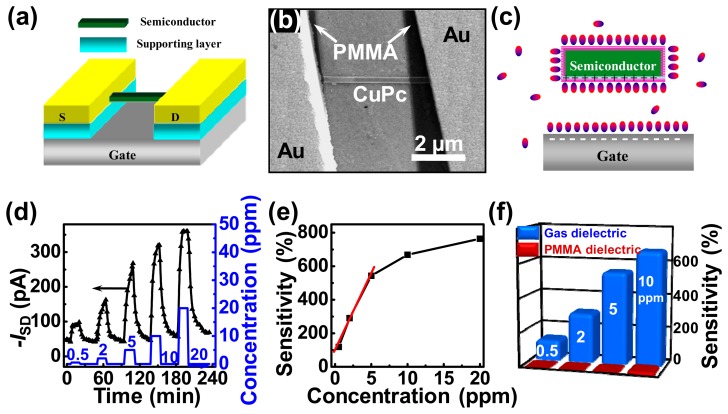
(**a**,**b**) The schematic and SEM images of gas dielectric FETs based on CuPc nanowire; (**c**) Schematic image for the work principle of gas dielectric sensor; (**d**) Real-time *I_SD_* change of device to various SO_2_ concentration (0.5, 2, 5, 10 and 20 ppm) at *V_G_* = −10 V and *V_SD_* = −15 V at room temperature. The blue line corresponds to SO_2_ concentration (right y-axis); (**e**) Sensitivity of device as a function of SO_2_ concentration at *V_G_* = −10 V and *V_SD_* = −15 V at room temperature. The red line is the linear fitting result in low-concentration SO_2_; (**f**) Comparison of the gas sensitivity of gas dielectric (blue) and PMMA dielectric (red) devices in different SO_2_ concentration (0.5, 2, 5 and 10 ppm) [5].

As mentioned in Section 3.1, in resistor sensors, the current passes through the whole semiconductor section which acts as the conductive channel. However, the conductive channel of the FETs is located in a few molecular layers at the interface between the semiconductor and the dielectric. Therefore, the gas adsorption in the conductive channel of FETs has a far larger influence than on the upper and side surface of the semiconductor layer. Further, for the traditional solid dielectric field-effect sensor, the conductive channel is capped by the semiconductor layer and the solid dielectric, and the gas molecules mainly adsorb to the upper and the two side surfaces of semiconductor (Figure 2). In contrast, the gas dielectric makes the conductive channel exposed to the detected gas (as shown in Figure 9c), which facilitates the direct interaction between the gas molecules and the conductive channel resulting in the improvement of the sensing performance.

Tang *et al.* demonstrated a gas dielectric field-effect sensor, where CuPc nanowire was used as semiconductor [5]. This is the first demonstration of SO_2_ gas sensing based on organic FETs. Figure 9a,b are the schematic and SEM images of the device. The individual CuPc nanowire bridged on the supporting layer, and the Au films were stamped on the side of the CuPc nanowire to form the source and drain electrodes. Their experiment results showed that the gas dielectric field-effect sensor presented excellent parameters such as sensitivity, LOD, response time, recovery time, resolution, and operating temperature. Some of them were comparable to the commercialized solid electrolyte sensors. Figure 9d,e present the current response and the sensitivity in different SO_2_ concentrations. The sensor was operated at room temperature. The LOD decreased over one order of magnitude (from 10 to 0.5 ppm) when the device configuration was changed from solid to gas dielectric. The LOD of gas dielectric sensor was 0.5 ppm with high sensitivity (119%) and high resolution (100 ppb), which is in a class with the highest detection limit for the semiconductor SO_2_ sensors reported so far. The gas dielectric sensor showed the sensitivity of about 800% in 30 ppm SO_2_, while the sensitivity of the solid dielectric sensor was only about 40% in the same SO_2_ concentration. The histogram in Figure 9f clearly presents the dramatically improved sensitivity in different SO_2_ concentration when the dielectric was changed from solid to gas state. The response and recovery times of the gas dielectric device in 0.5 ppm SO_2_ were only 3 and 8 min, respectively. The sensitivity showed the linear dependence on the concentration from 0.5 to 5 ppm with the change of 20% per 100 ppb SO_2_, corresponding to a concentration resolution of 100 ppb. It showed that the device was applicability for the detection of low-concentration SO_2_. The linear sensitivity dependence of Figure 9e in the low concentration (0–5 ppm) also showed the notable merits of the sensors for quantitative detection, direct electrical readout, and simplified device calibration process and auxiliary circuitry.

#### 3.4.2. Conductive Nanowires as Electrodes

**Figure 10.**
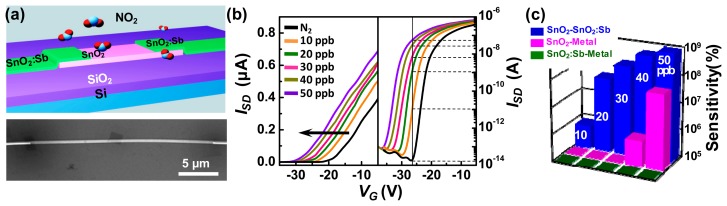
(**a**) Schematic and SEM image of the SnO_2_ nanobelt field-effect gas sensor with SnO_2_:Sb nanobelts as electrodes; (**b**) Transfer characteristics of the device to various NO_2_ concentrations (0, 10, 20, 30, 40 and 50 ppb) at room temperature; (**c**) Comparison of the sensitivity of the devices for different NO_2_ concentrations (10, 20, 30, 40 and 50 ppb): SnO_2_:Sb nanobelt with metal electrodes (green), SnO_2_ nanobelt FET with metal electrodes (red), and SnO_2_ nanobelt FET with SnO_2_:Sb electrodes (blue) [10].

The contact interface between the electrodes and the semiconductor also plays an important role in gas sensing. Barbara *et al.* found that the response was delayed when the CNT/electrode contacts were covered with a thick and long passivation layer [18]. Tang *et al.* employed a novel design, by introducing the high-conductivity SnO_2_:Sb nanobelts as electrodes, to fabricate the SnO_2_ nanobelt field-effect sensors [10]. Figure 10a shows the schematic and SEM images of the device. The SnO_2_:Sb nanobelt has the low resistivity (10^−2^–10^−4^ Ω·cm) and hence are capable to serve as the source and drain electrodes of the field-effect nanosensor. The good energy level matching of SnO_2_:Sb with SnO_2_ are also favorable for the carrier injection. The comparable size of the electrodes to the semiconductor also showed the potential application in nanoelectronics. Figure 10b is the transfer curves of the device for different NO_2_ concentrations. The LOD was shown to reach the ppb level (10 ppb), while maintaining both high sensitivity (7.16 × 10^5^%) and high resolution (−3.2 V per 10 ppb). Sensitivity value was as high as 1.92 × 10^8^% for NO_2_ at 30 ppb, which was six orders of magnitude higher than the previously reported highest sensitivity, for concentration below the 50 ppb level. In addition, the *V_T_* presented the linear dependence with the NO_2_ concentration. The LOD, sensitivity and resolution of the sensors were even superior to the commercially available MiCS-2714 metal oxide sensor (30% at 50 ppb) [60].

In their experiments, the transfer curves shifted towards the negative direction upon exposure to NO_2_, as shown in Figure 10b. Such a change suggested a sensing mechanism different from that of the extensively reported semiconductor sensors. They further measured the sensing performance of the SnO_2_ nanobelt field-effect sensor with the metal electrodes, and SnO_2_:Sb nanobelt resistor sensor with the metal electrodes. The comparative results are presented in Figure 10c. The SnO_2_:Sb nanobelt did not respond to NO_2_ in the concentration range of 10–50 ppb. The field-effect sensor with metal electrodes showed a sensitivity almost four orders of magnitude lower than the field-effect device with SnO_2_:Sb nanobelt electrodes in 10 ppb NO_2_. These comparative experiments showed that the interface between the SnO_2_ nanobelt and SnO_2_:Sb nanobelt electrodes was responsible for the sensing of devices to NO_2_.

#### 3.4.3. Nanowires Array as Semiconductor Layer

Multiple-nanowires or tube arrays as semiconductor layers of field-effect sensors accumulate the responses of the multiple wires, and hence are sensitive for gas detection. The multi-nanowire or tube averaging makes the electrical noise of the sensor far lower than that of the individual nanostructure device since the noise scales as 1/N (N is the number of tubes) [16,21]. Highly order and parallel array are requisite since the intersected network structure generally causes failed measurements in the field-effect properties due to the poor interface contact between semiconductor and dielectric.

Dai *et al.* employed a multiple CNT array as the semiconductor layer of a field-effect sensor [16]. The SWNT array was grown from the micropatterned catalyst on the preprepared Mo source and drain electrodes by chemical vapor deposition. The SWNT could adhere well onto the substrate surface. The average number of SWNTs across the electrodes for the single device was ∼20–30. Such a device has many advantages such as simple fabrication method, high yield, good electrical stability with low noise, and highly sensitivity. The individual nanotubes presented over 10% fluctuation of the conductance with time while the array device only showed ∼1% fluctuation. The electrical conductance of a typical MT device increased by 80% when was exposed to 100 ppb of NO_2_ in Ar.

**Figure 11.**
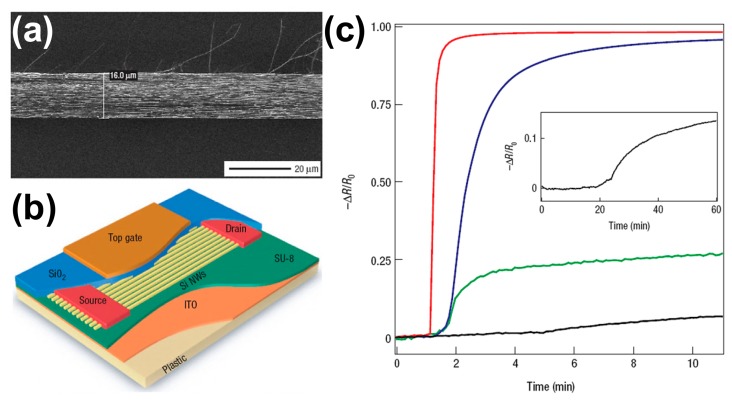
(**a**) SEM image of the transferred nanowires on plastic; (**b**) Schematic image of nanowire field-effect sensor on plastic; (**c**) Electrical response of a nanowire array sensor to 20 ppm (red curve), 2 ppm (blue curve), 200 ppb (green curve) and 20 ppb (black curve) NO_2_ diluted in N_2_. Inset: an extended response of the sensor to 20 ppb NO_2_ [21].

Heath *et al.* selected a Si nanowire array as the semiconductor layer for field-effect sensors [21]. The morphology of the nanowire array is shown in Figure 11a. Figure 11b is the schematic image of the nanowire array sensor. One of main advantages for Si nanowires is the precise control over the dopant type and concentration. The devices were fabricated on a bendable flexible plastic substrate. Figure 11c shows the normalized response of a nanowire sensing element to NO_2_ at different concentrations. The device exhibited sensitivities comparable to the best nanotube and metal-oxide nanowire devices on Si substrates [6,16,61]. The current increased ∼3000% after 20 ppm NO_2_ was introduced in N_2_ for 1.25 min. The LOD was at least 20 ppb of NO_2_ with 10% current increase after 15 min of exposure (Figure 11c, inset). They attributed this exquisite sensitivity to the residence of the charge carriers on the top surface of the nanowires.

## 4. Conclusions and Outlook

1D nanostructure field-effect sensors have obvious advantages of high sensitivity, rapid response, small size, room-temperature operation without heating sources, and compatibility with microelectronic integrated circuits. They provide unique amplification strategies to push the LOD to lower levels. More importantly, they provide a way to overcome the big challenge of semiconductor sensors in gas identification. Some efforts have been done for promoting the practical application of 1D nanostructure field-effect sensors, but only a few 1D nanostructure field-effect sensors have been reported until now, and some critical issues still need to be resolved as follows:

(i) Nanoscaled device fabrication methods.
  Most 1D nanostructure sensors apply a two-terminal resistor configuration, which has advantages of simple fabrication and facile measurements. The field-effect devices based on 1D nanostructures are difficult to produced due to the lack of the nanodevice fabrication techniques, which mainly include focused ion beam (FIB) and electron beam lithography (EBL).
(ii) Low-concentration detection.
  Although the LOD can be decreased by combining 1D nanostructures and the field-effect device configurations, the detectable concentration of the most reported sensors cannot meet the requirements of environmental monitoring. For example, NO_2_ and SO_2_ are two of the most dangerous environmental pollutants, and SO_2_ is difficult to detect with a semiconductor sensor. The U.S. Environmental Protection Agency has set the acceptable limit in ambient air at a level of 53 ppb for NO_2_ and 500 ppb for SO_2_, respectively [5,10]. Only a few sensors have reached such levels (Table 1).
(iii) Gas identification.
  Until now, how to achieve selective gas sensing in a highly complex background is still an unresolved problem. Future research should further develop the fabrication technique of the nanoscaled sensors, decrease the LOD of sensors, and improve the recognition capacity for practical applications. Other issues, for example, the standard definition of sensor parameters and testing method, long-term stabilities, as well as calibration of the sensors, should also be considered for the development of 1D nanostructure field-effect sensors.

## Figures and Tables

**Table 1.** The performance of reported 1D nanostructure field-effect sensors.

**Material**	**NW/NB/NT ^b^ case**	**Operating Temperature (°C)**	**Target**	**LOD ^e^**	**Sensitivity at LOD**	**Dimension (μm)**	**Response Time**	**Recover Time**	**Ref.**
In_2_O_3_	single	RT ^c^	NO_2_	500 ppb	*	0.01	10∼12 min	*	[3]
In_2_O_3_	single	RT	NH_3_	0.02%	*	0.01	2 min	*	[3]
In_2_O_3_	single	RT	NO_2_	20 ppb	25% ^g^	0.01	*	*	[6]
In_2_O_3_:Zn	single	RT	CO	1 ppm	*	0.125	20 s	10 s	[7]
In_2_O_3_:Mg	single	RT	CO	500 ppb	6.9 × 10^4^% ^1/f^	0.05	8 s	*	[8]
SnO_2_	single	100	H_2_	10 ppm	*	0.06	*	*	[9]
SnO_2_	single	RT	NO_2_	10 ppb	7.16 × 10^5^% ^f^	∼0.3	*	*	[10]
SnO_2_	single	250	H_2_S	500 ppm	150% ^f^	∼0.56	*	*	[11]
SnO_2_	single	250	CO	100 ppm	*	∼0.56	*	*	[11]
SnO_2_	single	250	CH_4_	100 ppm	*	∼0.56	*	*	[11]
SnO_2_:Sb	single	RT	ethanol	40 ppm	113% ^f^	0.05∼0.15	9 s	44 s	[12]
ZnO	single	RT	O_2_	10 ppm	64%^g^	0.06	*	*	[13]
ZnO	single	RT	NO_2_	200 ppb	*	*	*	*	[14]
CuO	single	200	CO	100 ppm	*	0.05∼0.1	10 s	*	[15]
CNT	multiple	RT	NO_2_	100 ppt	80% ^g^	0.002	*	*	[16]
CNT	single	RT	NO_2_	300 ppm	*	0.001	*	*	[17]
CNT	single	RT	NO_2_	40 ppm	3% ^f^	*	*	*	[18]
CNT	multiple	RT	DPCP ^d^	1 ppm	10^4^% ^f^	*	10 s	*	[19]
CNT	single	RT	NO_2_	2 ppm	*	∼0.0014	300 s	*	[20]
CNT	single	RT	NH_3_	0.02%	*	∼0.0014	600 s	*	[20]
Si	multiple	RT	NO_2_	20 ppb	13% ^g^	0.018	*	*	[21]
InAs	single	RT	IPA	*	*	0.025	*	*	[22]
InAs	single	RT	acetone	*	*	0.025	*	*	[22]
InAs	single	RT	ethanol	*	*	0.025	*	*	[22]
InAs	single	RT	H_2_O	*	*	0.025	*	*	[22]
CHICZ ^a^	single	RT	ethanol	*	*	3∼6	*	*	[23]
CuPc	single	RT	SO_2_	500 ppb	119% ^g^	0.21	3 min	8 min	[5]

a CHICZ: indolo[3,2-b]carbazole,2,8-dichloro-5,11-dihexylindolo[3,2-b] carbazole;

b NW/NB/NT: Nanowire/Nanobelt/Nanotube;

c RT: Room temperature;

d DPCP: (diphenylchlorophosphate)IPA(Isopropyl Alcohol);

e LOD: Limit of detection;

f *S = P_g_/P_a_ ×* 100*%*;

g *S = (P_g_ − P_a_)/P_a_ ×* 100%, *P_g_* is the test signal of the sensor in the target gas, *P_a_* is the test signal of the sensor in carrier gas.
